# Nonlinear association of triglyceride-glucose index with hyperuricemia in US adults: a cross-sectional study

**DOI:** 10.1186/s12944-024-02146-5

**Published:** 2024-05-17

**Authors:** Linjie Qiu, Yan Ren, Jixin Li, Meijie Li, Wenjie Li, Lingli Qin, Chunhui Ning, Jin Zhang, Feng Gao

**Affiliations:** 1 Xiyuan Hospital, China Academy of Chinese Medical Sciences, Beijing, China; 2 Shanxi University of Chinese Medicine, Taiyuan, Shanxi China

**Keywords:** Triglyceride-glucose index, Hyperuricemia, Cross-sectional study, NHANES, Nonlinear

## Abstract

**Background:**

Despite abundant evidence on the epidemiological risk factors of metabolic diseases related to hyperuricemia, there is still insufficient evidence regarding the nonlinear relationship between triglyceride-glucose (TyG) index and hyperuricemia. Thus, the purpose of this research is to clarify the nonlinear connection between TyG and hyperuricemia.

**Methods:**

From 2011 to 2018, a cross-sectional study was carried out using data from the National Health and Nutrition Examination Survey (NHANES). This study had 8572 participants in all. TyG was computed as Ln [triglycerides (mg/dL) × fasting glucose (mg/dL)/2]. The outcome variable was hyperuricemia. The association between TyG and hyperuricemia was examined using weighted multiple logistic regression, subgroup analysis, generalized additive models, smooth fitting curves, and two-piecewise linear regression models.

**Results:**

In the regression model adjusting for all confounding variables, the OR (95% CI) for the association between TyG and hyperuricemia was 2.34 (1.70, 3.21). There is a nonlinear and reverse U-shaped association between TyG and hyperuricemia, with a inflection point of 9.69. The OR (95% CI) before the inflection point was 2.64 (2.12, 3.28), and after the inflection point was 0.32 (0.11, 0.98). The interaction in gender, BMI, hypertension, and diabetes analysis was statistically significant.

**Conclusion:**

Additional prospective studies are required to corroborate the current findings, which indicate a strong positive connection between TyG and hyperuricemia among adults in the United States.

**Supplementary Information:**

The online version contains supplementary material available at 10.1186/s12944-024-02146-5.

## Introduction

Uric acid is produced when purine nucleotides are metabolized. The condition known as hyperuricemia occurs when uric acid levels rise over a certain point due to either excessive uric acid synthesis or inadequate uric acid elimination. It affects patients of all ages and genders, and its prevalence is on the rise globally [1, 2]. Up to 2016, the global prevalence of hyperuricemia has reached 21% [3], and the prevalence of hyperuricemia varies by geographic region. For example, in South Korea, it is 11.4% [4], and a survey conducted among adults aged 18–59 in China showed a prevalence of 15% for hyperuricemia [5]. Data from the 2007–2016 National Health and Nutrition Examination Survey (NHANES) show that 14.6–20% of Americans suffer with hyperuricemia [6]. Furthermore, hyperuricemia poses a serious threat to public health as numerous epidemiological studies have confirmed that it is a significant risk factor for a number of chronic diseases, including gout, cardiovascular diseases, chronic kidney disease, hypertension, metabolic syndrome, and many others [7–10], posing a serious threat to public health.

Insulin resistance (IR) is a physiological and pathological process closely associated with hyperuricemia [8]. Epidemiological studies have confirmed the close association between IR and serum urate concentration [11, 12]. High insulin levels induced by IR lead to reduced uric acid excretion and increased production, resulting in uric acid accumulation [13]. Reducing IR has been shown in studies to lower uric acid levels and lower the chance of developing gout [14]. An animal experimental study from Japan also found that insulin can promote uric acid reabsorption through urate transporter 1 and ATP-binding cassette sub-family G member 2 [15]. Additionally, a nationwide cohort study confirmed a significant association between insulin resistance and an increased risk of hyperuricemia [16]. When assessing IR, the glucose clamp method is regarded as the gold standard. However, the use of this detection technology in clinical practice is restricted because of its complexity and comparatively expensive cost. The body’s level of IR can be determined simply using the triglyceride-glucose (TyG) index [17]. The two main factors used to compute TyG are fasting triglycerides (TG) and fasting glucose (FPG). Multiple studies have confirmed its reliability in predicting various diseases related to IR [18–20]. TyG and hyperuricemia are significantly correlated in individuals with non-alcoholic fatty liver disease, diabetic nephropathy, and primary hypertension, according to earlier Chinese research [21–23]. Li et al. discovered that TyG might predict the coexistence of hypertension and hyperuricemia in the elderly population [24]. An additional cross-sectional study conducted in Northeast China examined the validity of TyG in determining the risk of hyperuricemia in people 40 years of age and older [25]. While prior research has indicated a connection between hyperuricemia and TyG index, these investigations mostly examined the Chinese population and had rather small sample sizes. The relationship between the TyG index and hyperuricemia is understudied in the US population. Wang et al. found a positive correlation between hyperuricemia and the TyG index in non-diabetic populations in the United States [26]. Furthermore, there are no reports on the relationship between the TyG index and hyperuricemia in the general adult population in the United States.

Therefore, for this cross-sectional analysis, NHANES data from 2011 to 2018 were used. This study aims to explore the relationship between TyG and adult Americans’ hyperuricemia.

## Methods

### Study design and population

This study made use of cross-sectional data from the National Center for Health Statistics (NCHS) 2011–2018 NHANES, a nationwide survey that used a sophisticated sampling design. The survey, conducted biennially since 1999, covers demographic, dietary, examination, laboratory, and questionnaire data [27, 28]. All participants completed informed permission forms, and the NHANES survey procedures and detailed data are available on the official website after being approved by NCHS.

**Fig. 1:**
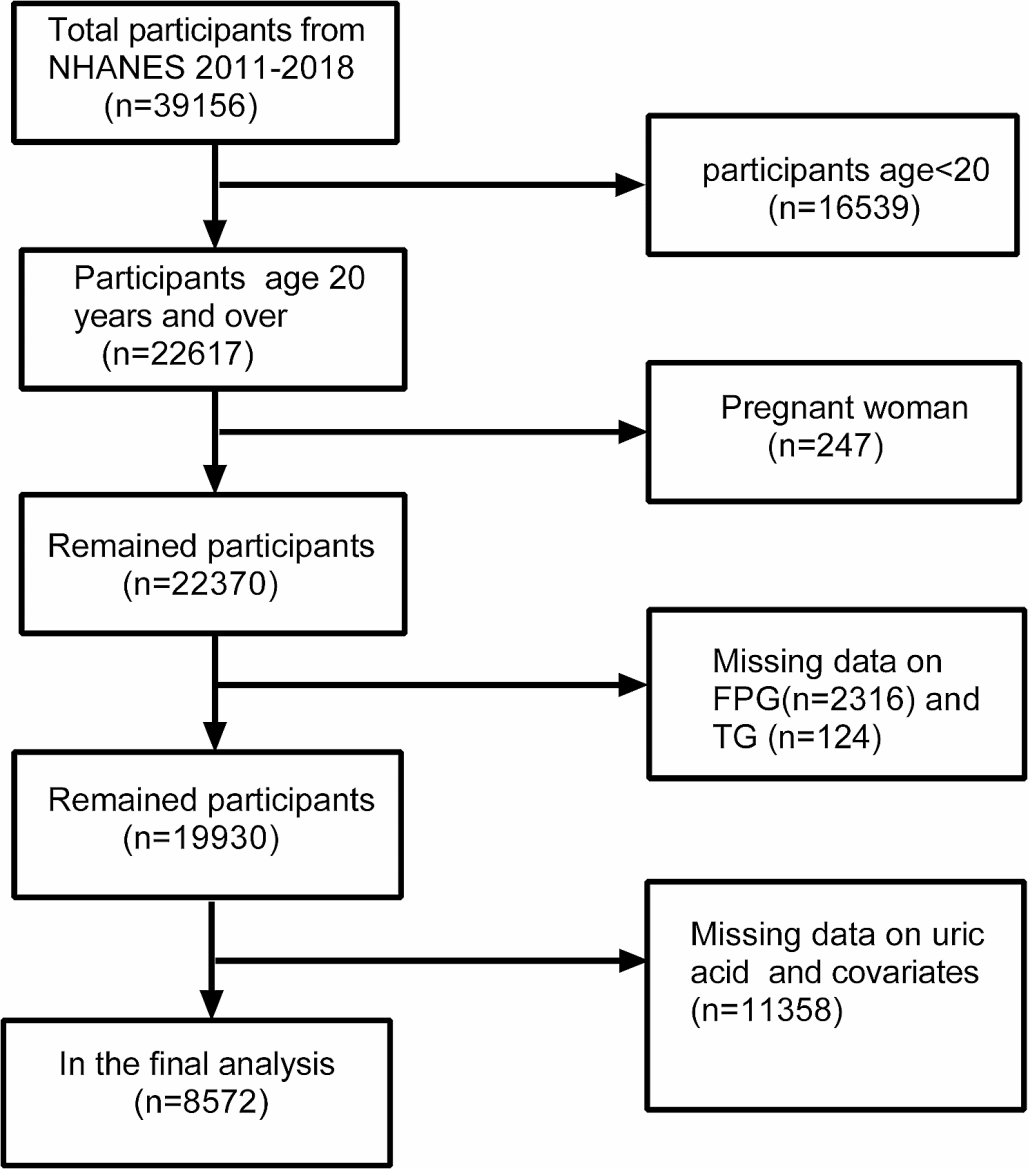
From chart of sample selection from the NHANES 2011–2018

After excluding 16,539 participants under the age of 20, 247 pregnant participants, 2,440 participants with missing FPG and TG data, and 11,358 participants with missing data on uric acid BMI, blood glucose, hypertension and related covariates, the final analysis includes 8,572 participants in total (Fig. 1).

### Definitions of the exposure and outcome variables

Employing an automatic analyzer, blood samples from individuals fasting for at least 8 h but less than 24 h were measured for TG and FPG using enzymatic methods. The TyG can be computed using the formula Ln [TG (mg/dL) × FPG (mg/dL)/2] [29]. By using uricase and H_2_O_2_ to undergo enzymatic oxidation, the concentration of uric acid in serum was determined and reported in milligrams per deciliter (mg/dL). This can be multiplied by 59.48 to get micromoles per liter (µmol/L). Serum uric acid levels ≥ 416 µmol/L (7 mg/dL) in men and ≥ 357 µmol/L (6 mg/dL) in women are classified as hyperuricemia, given the diagnostic criteria for the condition [30].

### Definition of covariates

To examine the distinct link between hyperuricemia and TyG, we adjusted for potential confounding factors, including demographics, lifestyle, anthropometric measurements, laboratory examinations, and health conditions. Age, gender, race, marital status, degree of education, and the ratio of household income to poverty were the main demographic factors; lifestyle mainly encompassed smoking status, alcohol consumption, and physical activity; anthropometric measurements primarily incorporated BMI; laboratory examination data mainly included HbA1c, LDL, HDL, eGFR, and serum creatinine; health conditions comprised hypertension, diabetes, arthritis, stroke, and coronary heart disease.

According to survey findings, “Yes” indicates that a person has smoked at least 100 cigarettes in their lifetime, whereas “no” indicates that they have smoked fewer than 100 [31]. Similarly, alcohol consumption is classified as “yes” (consuming at least 12 drinks per year) or “no” (consuming fewer than 12 drinks per year) [32]. Physical activity is grouped into three categories—active, moderately active, and inactive—following the guidelines for physical activity [33]. Three categories are used to classify education levels: below high school, high school, and above high school. Parameters such as HDL, LDL, HbA1c, and serum creatinine are measured from each participant’s fasting venous blood using an automated analyzer. Conditions like high blood pressure, heart disease, stroke, and arthritis are categorized based on self-reported medical diagnosis. The three factors used to identify diabetes are a self-reported medical diagnosis, a glycosylated hemoglobin (HbA1c) of 6.5% or above, or a fasting blood glucose level of 7.0 mmol/L or higher. The widely accepted algorithm developed by the Chronic Kidney Disease Epidemiology Collaboration is used to calculate the estimated glomerular filtration rate (eGFR) [34].

### Statistical analyses

Sample weights were appropriately applied in statistical analyses to account for complex sampling designs, following guidelines from the NHANES official website. All of the study population’s descriptive statistics were calculated, and the TyG index was divided into quartiles. The categorical data were reported as percentages, and the continuous variables were shown as mean ± standard deviation (SD). To examine differences between continuous and categorical data, weighted chi-square tests and weighted linear regression models were employed, respectively. In accordance with the STROBE statement [35], three distinct multivariate logistic regressions were run to investigate the relationship between TyG and hyperuricemia. While Model 2 and Model 3 adjusted for age, gender, and race, educational level, marital status, RIP, smoking, alcohol consumption, physical activity, BMI, HDL, LDL, HbA1c, serum creatinine, eGFR, hypertension, diabetes, arthritis, stroke, and coronary heart disease, Model 1 left covariates unadjusted. Relationship consistency was verified by a linear trend test, and nonlinear relationships were investigated using a Generalized Additive Model (GAM) with smooth curve fitting. In the presence of nonlinearity, a recursive algorithm identified significant turning points in the TyG and hyperuricemia relationship. Threshold effect analysis assessed differences between logistic regression models and two-part logistic regression models. Additionally, subgroup analyses and interaction tests were performed for age, gender, BMI, hypertension, diabetes, stroke, arthritis, and coronary heart disease, with adjustments for corresponding confounding factors. The results were considered credible if the interaction *P*-value was not significant; if it was, then likely subgroup variations were considered. EmpowerStats (http://www.empowerstats.com) and R (version 4.2.2) were used for all statistical analyses, with a *P*-value < 0.05 denoting statistical significance.

## Results

### Baseline characteristics of participants

Table 1 displays the baseline attributes of the individuals in the TyG index. Compared to the lowest TyG quartile, individuals in the TyG Q4 group exhibited a tendency towards older age, male gender, Mexican American ethnicity, lower educational attainment, marital status, non-smoking behavior, lower RIP levels, lower HDL, lower eGFR, and higher prevalence of hypertension, diabetes, coronary heart disease, arthritis, stroke. Additionally, they displayed higher levels of BMI, HbA1c, FPG, TG, LDL, serum creatinine, and uric acid (all *P* < 0.05). Notably, there was a significantly increased frequency of hyperuricemia (*P* < 0.05) in participants with high TyG levels.

**Table 1:** Baseline characteristics of the study population according to the quartiles of the TyG index

	Q1, *N* = 2141	Q2, *N* = 2145	Q3, *N* = 2143	Q4, *N* = 2143	*P*-value
TyG index	7.86 ± 0.27	8.44 ± 0.13	8.89 ± 0.14	9.70 ± 0.50	< 0.001
Age(years)	42.70 ± 17.48	49.08 ± 17.95	51.67 ± 16.90	53.34 ± 15.80	< 0.001
Gender (%)					< 0.001
Male	896 (41.85%)	1042 (48.58%)	1096 (51.14%)	1246 (58.14%)	
Female	1245 (58.15%)	1103 (51.42%)	1047 (48.86%)	897 (41.86%)	
Race (%)					< 0.001
Mexican American	180 (8.41%)	253 (11.79%)	298 (13.91%)	372 (17.36%)	
Other Hispanic	163 (7.61%)	213 (9.93%)	247 (11.53%)	258 (12.04%)	
Non-Hispanic White	742 (34.66%)	854 (39.81%)	890 (41.53%)	846 (39.48%)	
Non-Hispanic Black	738 (34.47%)	529 (24.66%)	405 (18.90%)	305 (14.23%)	
Non-Hispanic Asian	239 (11.16%)	219 (10.21%)	232 (10.83%)	289 (13.49%)	
Other Race	79 (3.69%)	77 (3.59%)	71 (3.31%)	73 (3.41%)	
Educational level (%)					< 0.001
< High school	347 (16.21%)	426 (19.86%)	460 (21.47%)	550 (25.66%)	
High school	449 (20.97%)	507 (23.64%)	490 (22.87%)	494 (23.05%)	
> High school	1345 (62.82%)	1212 (56.50%)	1193 (55.67%)	1099 (51.28%)	
Marital status (%)					< 0.001
Married	886 (41.38%)	1043 (48.62%)	1144 (53.38%)	1199 (55.95%)	
Widowed	111 (5.18%)	164 (7.65%)	173 (8.07%)	173 (8.07%)	
Divorced	200 (9.34%)	227 (10.58%)	237 (11.06%)	259 (12.09%)	
Separated	83 (3.88%)	70 (3.26%)	73 (3.41%)	78 (3.64%)	
Never married	657 (30.69%)	454 (21.17%)	341 (15.91%)	268 (12.51%)	
Living with partner	204 (9.53%)	187 (8.72%)	175 (8.17%)	166 (7.75%)	
Smoking status (%)					< 0.001
No	1345 (62.82%)	1241 (57.86%)	1198 (55.90%)	1103 (51.47%)	
Yes	796 (37.18%)	904 (42.14%)	945 (44.10%)	1040 (48.53%)	
Alcohol consumption (%)					0.113
No	822 (38.39%)	890 (41.49%)	882 (41.16%)	841 (39.24%)	
Yes	1319 (61.61%)	1255 (58.51%)	1261 (58.84%)	1302 (60.76%)	
Physical activity (%)					0.470
Inactive	1232 (57.54%)	1257 (58.60%)	1271 (59.31%)	1306 (60.94%)	
Less active	168 (7.85%)	169 (7.88%)	167 (7.79%)	160 (7.47%)	
Namely active	741 (34.61%)	719 (33.52%)	705 (32.90%)	677 (31.59%)	
RIP	2.60 ± 1.68	2.59 ± 1.64	2.52 ± 1.63	2.41 ± 1.61	< 0.001
BMI (kg/m^2^)	26.84 ± 6.83	28.93 ± 7.11	30.22 ± 6.92	31.44 ± 6.70	< 0.001
HbA1c (%)	5.40 ± 0.59	5.55 ± 0.61	5.73 ± 0.80	6.47 ± 1.70	< 0.001
FPG (mmol/l)	4.87 ± 0.66	5.23 ± 0.86	5.55 ± 1.22	7.36 ± 3.72	< 0.001
TG (mmol/l)	0.70 ± 0.18	1.14 ± 0.20	1.72 ± 0.36	3.36 ± 2.00	< 0.001
LDL (mmol/l)	2.60 ± 0.75	2.97 ± 0.87	3.11 ± 0.97	3.02 ± 1.05	< 0.001
HDL (mmol/l)	1.62 ± 0.43	1.45 ± 0.39	1.31 ± 0.34	1.10 ± 0.28	< 0.001
Serum creatinine (umol/L)	77.64 ± 42.38	79.33 ± 42.32	80.22 ± 29.08	83.95 ± 49.28	< 0.001
Uric acid (umol/L)	295.90 ± 76.44	318.10 ± 80.52	333.67 ± 84.16	349.02 ± 87.21	< 0.001
eGFR (ml/min per 1.73m^2^)	108.82 ± 31.88	102.87 ± 32.19	99.21 ± 31.14	96.40 ± 32.55	< 0.001
Hypertension (%)					< 0.001
No	1604 (74.92%)	1458 (67.97%)	1257 (58.66%)	1130 (52.73%)	
Yes	537 (25.08%)	687 (32.03%)	886 (41.34%)	1013 (47.27%)	
Diabetes (%)					< 0.001
No	2032 (94.91%)	1926 (89.79%)	1774 (82.78%)	1269 (59.22%)	
Yes	109 (5.09%)	219 (10.21%)	369 (17.22%)	874 (40.78%)	
Arthritis (%)					< 0.001
No	2032 (94.91%)	1926 (89.79%)	1774 (82.78%)	1269 (59.22%)	
Yes	109 (5.09%)	219 (10.21%)	369 (17.22%)	874 (40.78%)	
Coronary heart disease (%)					< 0.001
No	2086 (97.43%)	2081 (97.02%)	2054 (95.85%)	2023 (94.40%)	
Yes	55 (2.57%)	64 (2.98%)	89 (4.15%)	120 (5.60%)	
Stroke (%)					< 0.001
No	2089 (97.57%)	2070 (96.50%)	2059 (96.08%)	2061 (96.17%)	
Yes	52 (2.43%)	75 (3.50%)	84 (3.92%)	82 (3.83%)	
Hyperuricemia (%)					< 0.001
No	1933 (90.28%)	1799 (83.87%)	1715 (80.03%)	1590 (74.20%)	
Yes	208 (9.72%)	346 (16.13%)	428 (19.97%)	553 (25.80%)	

Mean ± SD for continuous variables: *P* value was calculated by weighted linear regression model. % for categorical variables: *P* value was calculated by weighted chi-square test

BMI body mass index, RIP ratio of family income to poverty, LDL-C low-density lipoprotein cholesterol, HDL-C high-density lipoprotein cholesterol, HbA1c glycohemoglobin, FPG fasting plasma glucose, TG triglycerides, eGFR glomerular filtration rate, TyG index triglyceride-glucose index

### Association between TyG and its components and hyperuricemia

Table 2 displays the relationship between TyG and its components and hyperuricemia. After adjusting for potential confounding variables (Model 3), the study found a significant positive correlation between TG and hyperuricemia (OR = 1.68, 95% CI: 1.38, 2.04). Further dividing TG into quartiles, in Model 3, participants in the highest quartile of TG had a 1.95-fold higher risk of hyperuricemia compared to those in the lowest quartile (OR: 2.95, 95% CI: 1.83, 4.75). Additionally, a significant dose-response relationship was found (*P* < 0.05). However, after adjusting for potential confounding variables (Model 3), the study did not find a significant association between FPG and hyperuricemia (OR = 1.00, 95% CI: 0.99, 1.01). Further dividing FPG into quartiles, in Model 3, participants in quartile 4 of FPG had a significantly positive correlation with hyperuricemia compared to Q1 (OR = 1.84, 95% CI: 1.14, 2.99). Our study also found a significant dose-response relationship (*P* < 0.05). Moreover, the investigation’s findings demonstrated a positive correlation between TyG and the likelihood of hyperuricemia. Variable adjustments bolstered this association, and all multivariate logistic regression models (model 1: OR = 1.70, 95% CI: 1.51,1.91; model 2: OR = 1.69, 95% CI: 1.50,1.92; model 3: OR = 2.34, 95% CI: 1.70,3.21) showed positive correlations regardless of whether confounding variables were adjusted. It’s interesting to note that a unit increase in the TyG index was linked to a 1.34-fold increase in the risk of hyperuricemia after controlling for possible confounding variables (model 3; Table 2). When TyG was further split into quartiles using Q1 as the reference group and different variables were taken into account in model 3, the risk of hyperuricemia was 3.85 times higher for those in the highest quartile of the TyG index than for those in the lowest quartile (OR: 4.85, 95% CI: 3.03, 7.78) (Table 2). Furthermore, a noteworthy dose-response correlation (*P* < 0.05) was noted.

**Table 2:** Association between TyG and its components and hyperuricemia

OR (95%CI) *P*-value	Model 1	Model 2	Model 3
Continuous TG	1.15(1.05, 1.27) 0.005	1.15(1.04, 1.28) 0.009	1.68(1.38, 2.04) < 0.001
TG quartiles			
Q1	Reference	Reference	Reference
Q2	1.40(1.11, 1.77) 0.006	1.41(1.11, 1.79) 0.007	1.07(0.74, 1.53) 0.732
Q3	2.04(1.61, 2.59) < 0.001	2.05(1.61, 2.62) < 0.001	1.88(1.26, 2.80) 0.004
Q4	2.70(2.08, 3.51) < 0.001	2.73(2.07, 3.60) < 0.001	2.95(1.83, 4.75) < 0.001
*P* for trend	< 0.001	< 0.001	< 0.001
Continuous FPG	1.00(1.00, 1.01) < 0.001	1.00(1.00, 1.00) 0.004	1.00(0.99, 1.01) 0.876
FPG quartiles			
Q1	Reference	Reference	Reference
Q2	1.25(0.99, 1.57) 0.062	1.22(0.97, 1.54) 0.097	0.69(0.43, 1.11) 0.135
Q3	1.62(1.25, 2.09) < 0.001	1.51(1.16, 1.96) 0.004	0.93(0.61, 1.43) 0.745
Q4	2.52(2.04, 3.12) < 0.001	2.27(1.79, 2.87) < 0.001	1.84(1.14, 2.99) 0.020
*P* for trend	< 0.001	< 0.001	0.005
Continuous TyG	1.70(1.51, 1.91) < 0.001	1.69 (1.50, 1.92) < 0.001	2.34 (1.70, 3.21) < 0.001
TyG quartiles			
Q1	Reference	Reference	Reference
Q2	1.61 (1.26, 2.06) < 0.001	1.62 (1.26, 2.08) < 0.001	1.54 (0.98, 2.14) 0.072
Q3	2.22 (1.74, 2.83) < 0.001	2.22(1.72, 2.86) < 0.001	2.17 (1.44, 3.25) < 0.001
Q4	3.46 (2.67, 4.49) < 0.001	3.45 (2.62, 4.55) < 0.001	4.85 (3.03, 7.78) < 0.001
*P* for trend	< 0.001	< 0.001	< 0.001

Model 1:no covariates were adjusted

Model 2: age, sex and race were adjusted

Model 3: age, sex, race, educational level, marital status, smoking status, alcohol consumption, physical activity, BMI, RIP, LDL, HDL, HbA1c, Serum creatinine, eGFR, hypertension, diabetes, arthritis, coronary heart disease and Stroke were adjusted

**Fig. 2:**
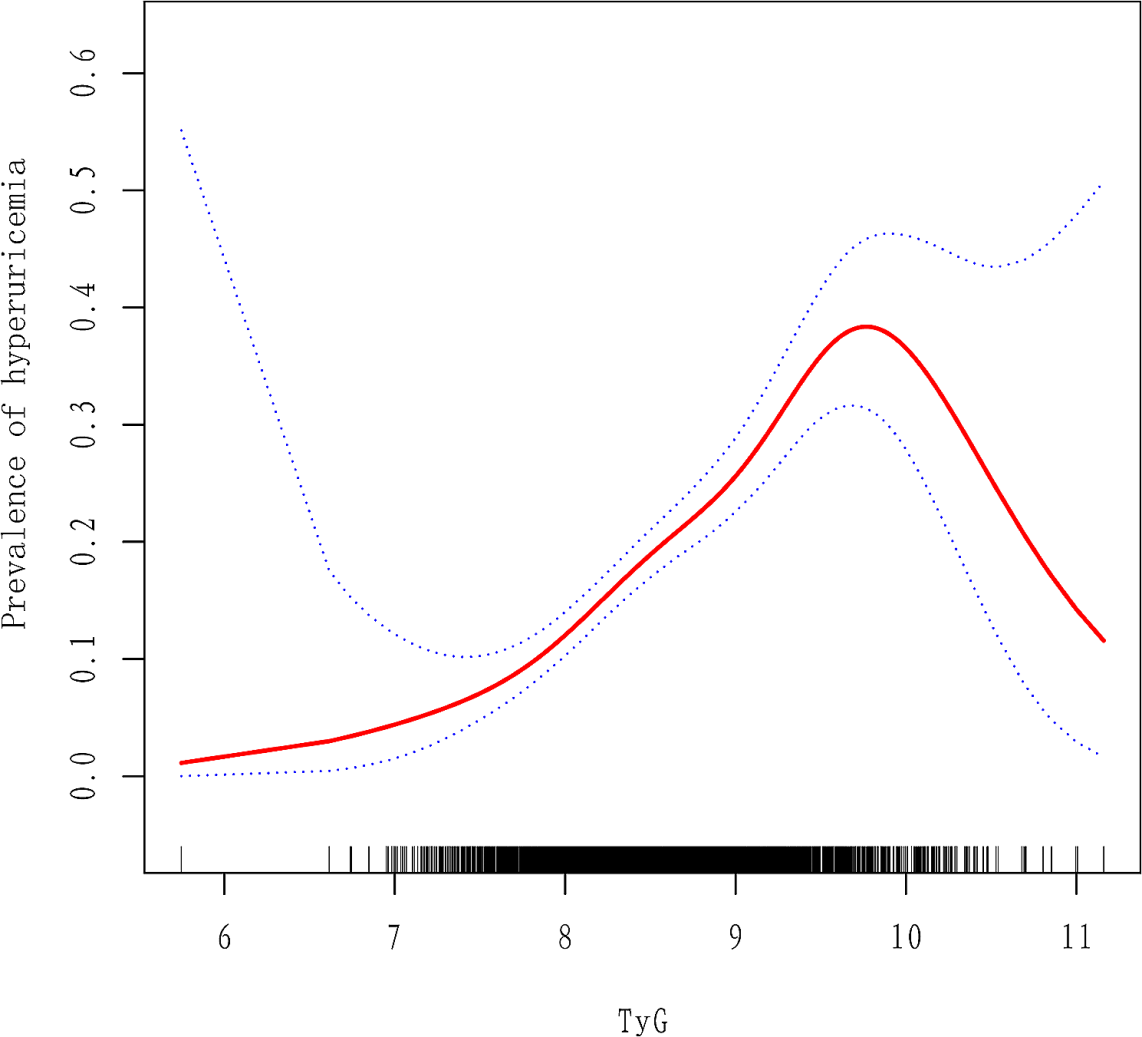
Smooth curve fitting for TyG and hyperuricemia. Non-linear relationship between TyG and hyperuricemia was detected by the generalized additive model. The solid red line represents the smooth curve fit between variables. Blue dotted lines represent the 95% CI from the fit. Adjustment factors included age, sex, race, educational level, marital status, smoking status, alcohol consumption, physical activity, BMI, RIP, LDL, HDL, HbA1c, Serum creatinine, eGFR, hypertension, diabetes, arthritis, coronary heart disease and Stroke

**Fig. 3:**
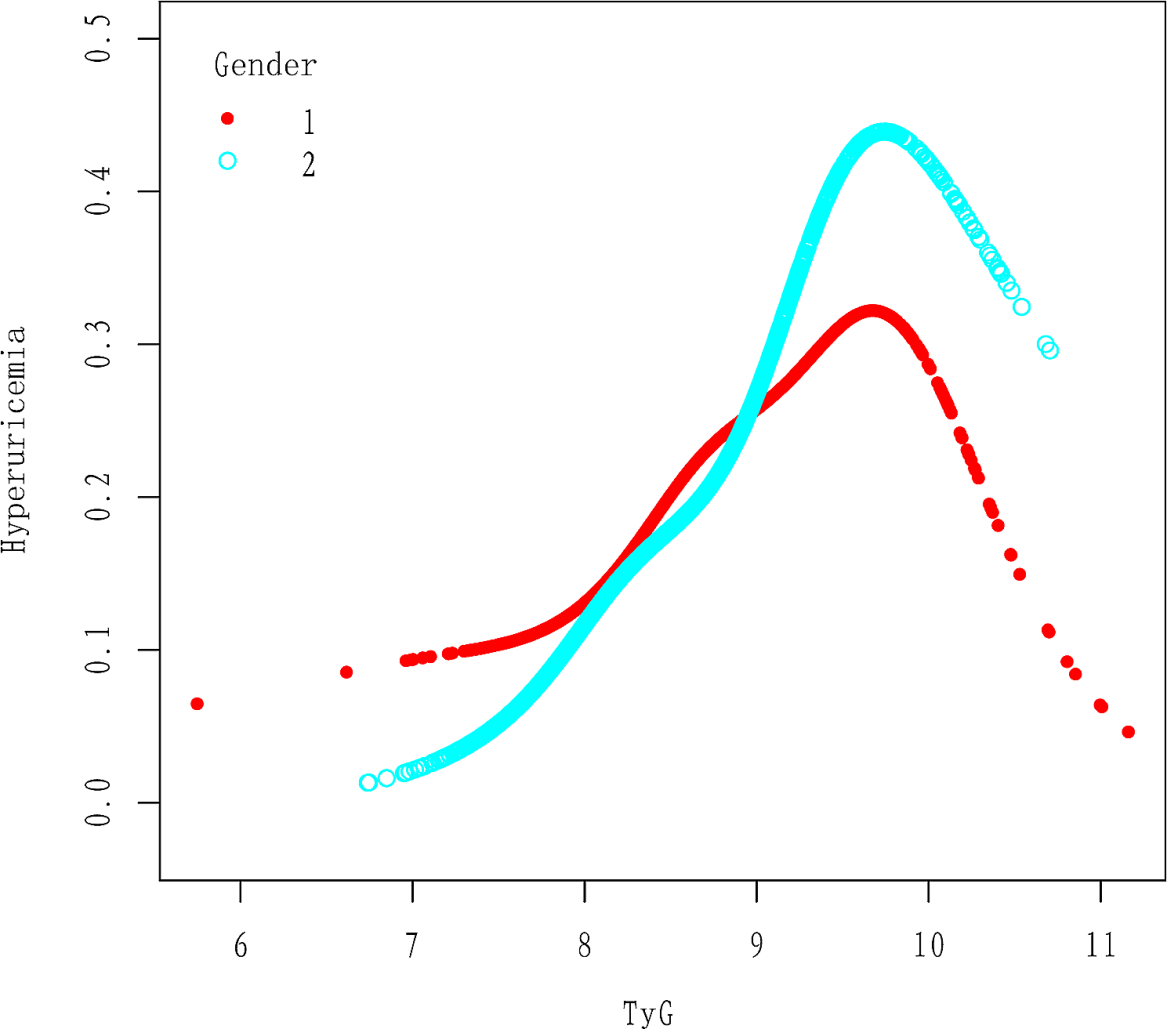
The association between TyG and hyperuricemia stratified by gender. Age, race, educational level, marital status, smoking status, alcohol consumption, physical activity, BMI, RIP, LDL, HDL, HbA1c, Serum creatinine, eGFR, hypertension, diabetes, arthritis, coronary heart disease and Stroke were adjusted

**Fig. 4:**
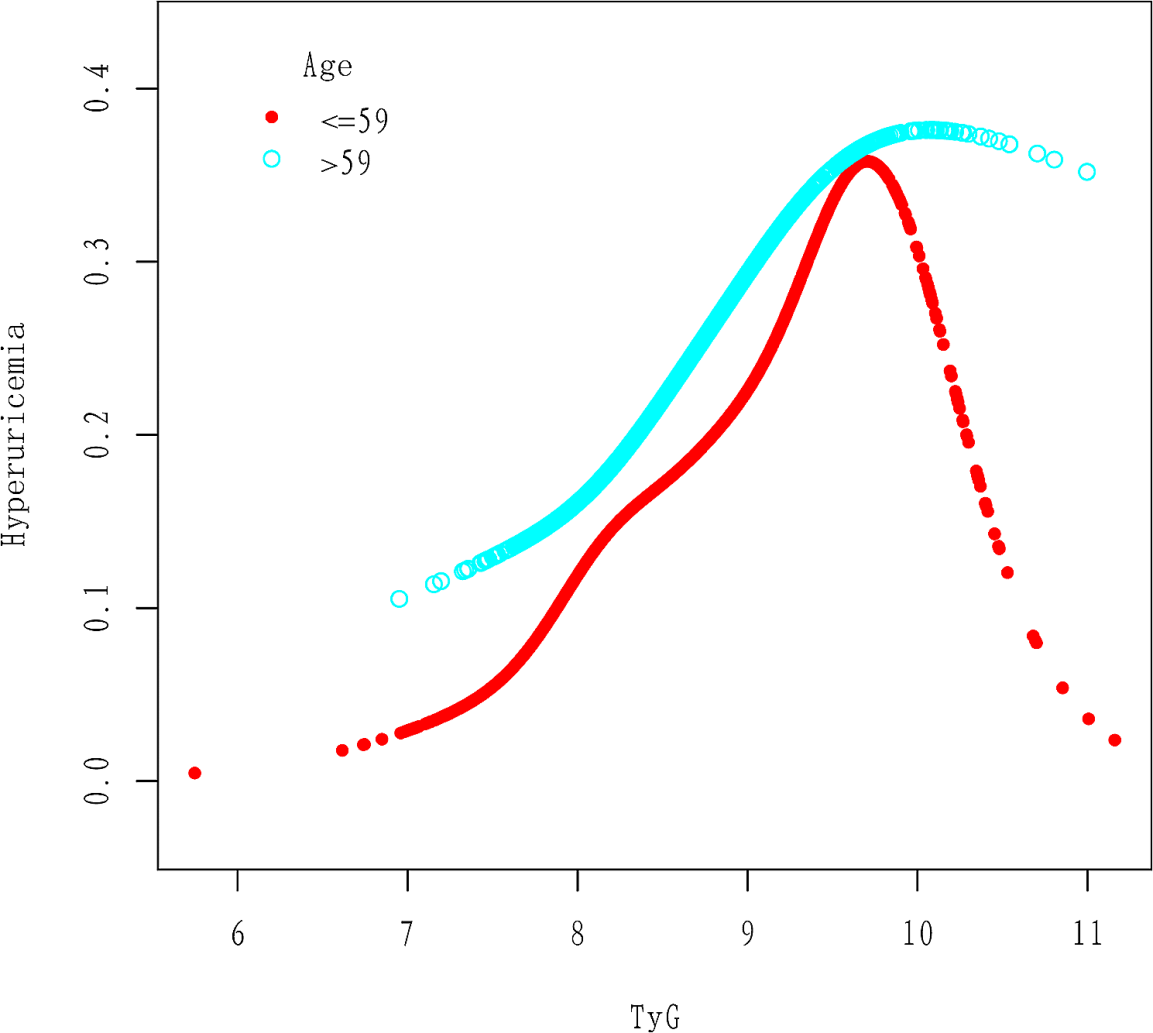
The association between TyG and hyperuricemia stratified by age. Gender, race, educational level, marital status, smoking status, alcohol consumption, physical activity, BMI, RIP, LDL, HDL, HbA1c, Serum creatinine, eGFR, hypertension, diabetes, arthritis, coronary heart disease and Stroke were adjusted

However, the odds ratios (ORs) for Q2, Q3, and Q4 show that there might be a non-linear correlation; the 95% confidence intervals (CIs) for these three questions are 1.54 (0.98, 2.14), 2.17 (1.44, 3.25), and 4.85 (3.03, 7.78), respectively. Using GAM and smooth curve fitting, a non-linear association between TyG and hyperuricemia was found (Fig. 2), adding to the validity of the results. Further exploration through threshold effect analysis revealed a turning point at 9.69 (Table 3). Before the turning point, TyG and hyperuricemia exhibited a significant positive correlation, with an OR (95% CI) of 2.64 (2.12, 3.28). Subsequently, after the turning point, TyG and hyperuricemia showed a significant negative correlation, with an OR (95% CI) of 0.32 (0.11, 0.98) (Table 3). Additionally, after stratification by age and gender, our results also indicate a non-linear relationship between TyG and hyperuricemia (Figs. 3 and 4).

**Table 3:** Threshold effect analysis of TyG on hyperuricemia using a two-piecewise linear regression model

Hyperuricemia	Adjust OR (95% CI)	*P*
TyG		
Fitting by standard linear model	2.25(1.84,2.75)	< 0.001
Fitting by two-piecewise linear model		
Inflection point	9.69	
< 9.69	2.64(2.12,3.28)	< 0.001
> 9.69	0.32(0.11,0.98)	0.046
Log-likelihood ratio	< 0.001	

age, sex, race, educational level, marital status, smoking status, alcohol consumption, physical activity, BMI, RIP, LDL, HDL, HbA1c, Serum creatinine, eGFR, hypertension, diabetes, arthritis, coronary heart disease and Stroke were adjusted

**Fig. 5:**
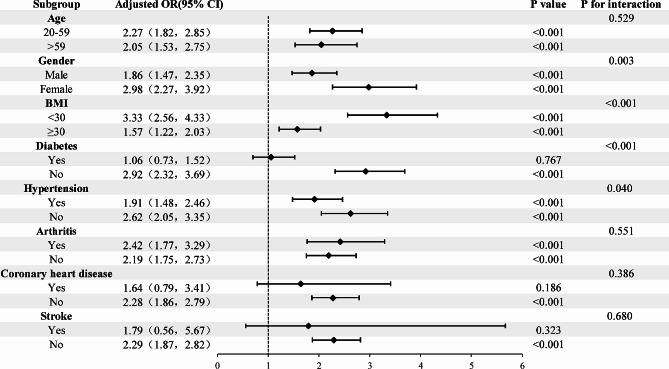
Subgroup and interaction analyses of the TyG index and hyperuricemia. Adjustment factors included age, sex, race, educational level, marital status, smoking status, alcohol consumption, physical activity, BMI, RIP, LDL, HDL, HbA1c, Serum creatinine, eGFR, hypertension, diabetes, arthritis, coronary heart disease and Stroke

To further evaluate the association between TyG and hyperuricemia in various categories, we also conducted interaction tests and stratified analysis accounting for gender, age, BMI, hypertension, diabetes, coronary heart disease, arthritis, and stroke. The positive link between TyG and hyperuricemia does not appear to be influenced by age, arthritis, coronary heart disease, or stroke, according to the results of our study. However, interactions were observed in gender, BMI, diabetes, and hypertension, with particular significance in female, non-obese, non-hypertensive, and non-diabetic populations (OR: 2.98, 95% CI: 2.27, 3.92), (OR: 3.33, 95% CI: 2.56, 4.33), (OR: 2.62, 95% CI: 2.05, 3.35), (OR: 2.92, 95% CI: 2.32, 3.69) (Fig. 5). Therefore, we further explored the non-linear relationship between TyG and hyperuricemia through stratification. After stratification by gender, we found that their non-linear relationship still exists (Fig. 3). Furthermore, after stratification by BMI, hypertension, and diabetes, we still observed a non-linear association (Supplementary Figs. 1, 2 and 3).

## Discussion

Based on NHANES data from 2011 to 2018, our large-sample cross-sectional analysis demonstrates a strong correlation between elevated TyG and a higher risk of hyperuricemia. Even when categorizing the TyG into quartiles (Q1-Q4), this positive correlation persists. In the adult population in the United States, we found a non-linear association between hyperuricemia and the TyG index after applying a smooth curve. There is a segmented inhibitory effect between the TyG index and hyperuricemia, with 9.69 as a significant inflection point. Before this point, a significant increase in hyperuricemia risk was reported with the increasing TyG, and after this point, a significant decrease in hyperuricemia risk was observed with increasing TyG index. Additionally, our study presents the most detailed stratified analysis.

The TyG index and hyperuricemia had a linear positive connection, according to a prior cross-sectional study from northeastern China, with a 54.1% rise in the probability of hyperuricemia for every unit increase in the TyG [25]. A cross-sectional study conducted in Thailand also found that among Royal Thai Army members, the TyG index and hyperuricemia had a substantial positive connection that persisted regardless of the soldiers’ obesity condition [36]. Qing et al. evaluated the relationship between TyG and hyperuricemia in a cohort study involving 42,387 Chinese patients having physical exams. The findings demonstrated a favorable relationship between hyperuricemia and the TyG index [37]. These studies support our findings. In addition, our research revealed a strong positive association between TyG and hyperuricemia, with each unit rise in TyG associated with a 1.34-fold increase in the risk of hyperuricemia. It was also discovered that interactions occurred regardless of obesity, however in non-obese people this link was stronger.

In addition, after conducting subgroup analyses and interaction tests, our study found that gender, hypertension, and diabetes interacted with the relationship between TyG and hyperuricemia, especially in females, and this association was more pronounced in non-hypertensive and non-diabetic populations. Gender variations have been observed in the TyG index’s ability to detect hyperuricemia in the past, particularly in females [38], which is consistent with our study results. This may be because estrogen is a uric acid generator and is associated with complex metabolic endocrine factors, thereby affecting lipid metabolism and causing gender differences in lipid metabolism [39]. In hypertensive people with an average age of 63.81 years, a study in China demonstrated a positive connection between TyG and hyperuricemia (OR = 2.04; 95%CI: 1.87 to 2.24) [40]. An additional cross-sectional study conducted in Chinese hospitals investigated the relationship between hyperuricemia and TyG in patients with hypertension. TyG and hyperuricemia were shown to positively correlate in hypertensive individuals; this correlation was more pronounced in patients with grade 1–2 hypertension than in those with grade 3 hypertension [22]. This is consistent with the trend observed in our study. Regardless of the existence of hypertension, we discovered a favorable connection between TyG and hyperuricemia, but this correlation was more pronounced in non-hypertensive individuals. Differences in demographic characteristics and research methods may explain this discrepancy. Further research is needed to uncover these underlying factors. Through a retrospective analysis, Han et al. [41] discovered a substantial positive connection between TyG and hyperuricemia in patients with diabetes, whereas our study discovered an interaction between TyG and hyperuricemia in patients without diabetes. The observed occurrence could potentially be attributed to variations in the study population, ethnicity, and sample size. More study is required to validate these findings because there is a dearth of information regarding the connection between TyG and hyperuricemia in both diabetic and non-diabetic groups.

The mechanism of TyG in hyperuricemia is not yet clear, but the following biological mechanisms can be explained. Since TyG is computed by summing up TG and FPG, there is a strong correlation between the pathophysiology of hyperuricemia and TG and FPG levels in the human body. Abnormalities in lipid metabolism result from the breakdown of elevated quantities of TG into free fatty acids, which are then transferred to different parts of the body and speed up the breakdown of adenosine triphosphate. Lipid metabolism abnormalities cause kidney damage, reduce uric acid excretion, and consequently increase serum uric acid levels [42]. Furthermore, high TG levels inhibit insulin receptor activity and quantity on adipocytes, competing with glucose to block insulin’s ability to bind to receptors and cause IR [43]. Excessive accumulation of glucose leads to hyperglycemia, alters the expression and activity of glucose transporter proteins in tissues, and reduces insulin sensitivity [44, 45]. Notably, with an inflection point of 9.69, our study discovered a strong segmental inhibitory effect between TyG and hyperuricemia. TyG and hyperuricemia had a substantial positive correlation up to 9.69, whereas a significant negative correlation followed after 9.69. This differs from the results reported in previous studies, and one possible reason is speculated to be racial differences. Previous correlation studies have mainly focused on Asian countries such as China and Thailand, and racial differences have been shown to affect insulin sensitivity [46]. Also differences in demographic characteristics and research methods may be potential factors. To sum up, additional pertinent research is required to validate our findings, particularly in the US population.

There are various restrictions on this study. First off, because the study is cross-sectional, we are unable to determine if TyG and hyperuricemia are causally related. The conclusions reached must be supported by further research. Second, although we included many relevant covariates and adjusted accordingly, there may still be interference from other confounding factors, such as hyperthyroidism, alcoholism, renal insufficiency, drugs, tumors, and other factors that affect uric acid levels. To substantiate the connection between hyperuricemia and the TyG index, more intervention studies ought to be carried out. Additionally, serum uric acid levels are influenced by diets rich in purines, and the data on dietary questionnaires in NHANES are very limited, so we cannot determine whether participants have a high-purine diet.

## Conclusion

In general, hyperuricemia and the TyG index have a reverse U-shaped connection. In patients with TyG < 9.69, a higher risk of hyperuricemia is significantly correlated with a greater TyG. On the other hand, a higher TyG is substantially linked to a decreased risk of hyperuricemia in patients with TyG > 9.69. These results imply that the prevention and treatment of hyperuricemia may benefit from reducing or raising TyG levels within a specific range. Confirming the causal relationship and underlying mechanisms between them will require more investigation.

### Electronic supplementary material

Below is the link to the electronic supplementary material.

Supplementary Material 1

Supplementary Material 2

Supplementary Material 3

Supplementary Material 4

## Data Availability

Data is provided within the supplementary information files.
