# Supplementary figures and images for: Nonlinear association of triglyceride-glucose index with hyperuricemia in US adults: a cross-sectional study
Source: Lipids Health Dis. 2024 May 17;23:145. doi: 10.1186/s12944-024-02146-5 (PMC11100171; doi:10.1186/s12944-024-02146-5)

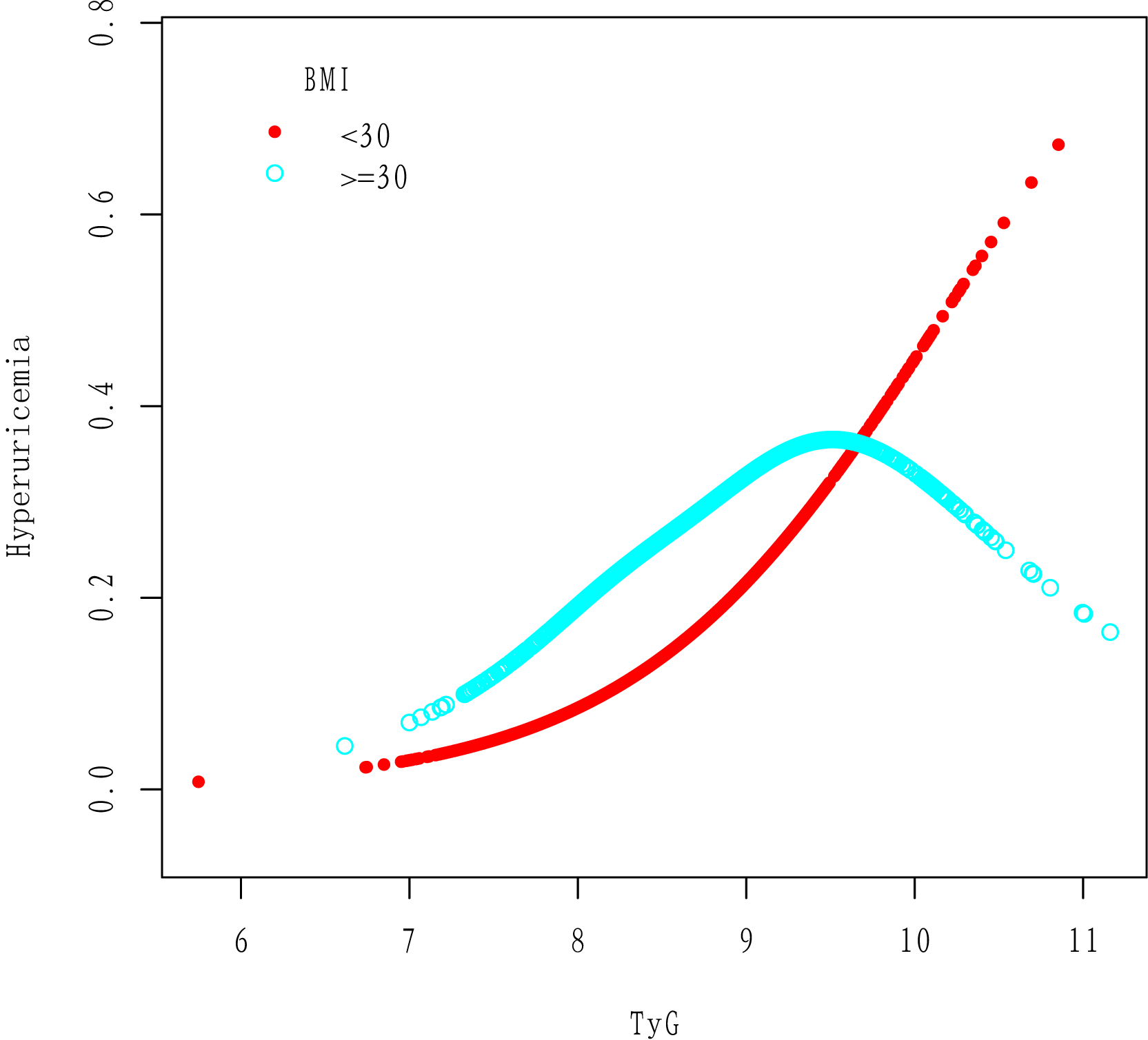

Supplement: Supplementary file 2 — Supplementary Material 2 [file 12944_2024_2146_MOESM2_ESM.png]

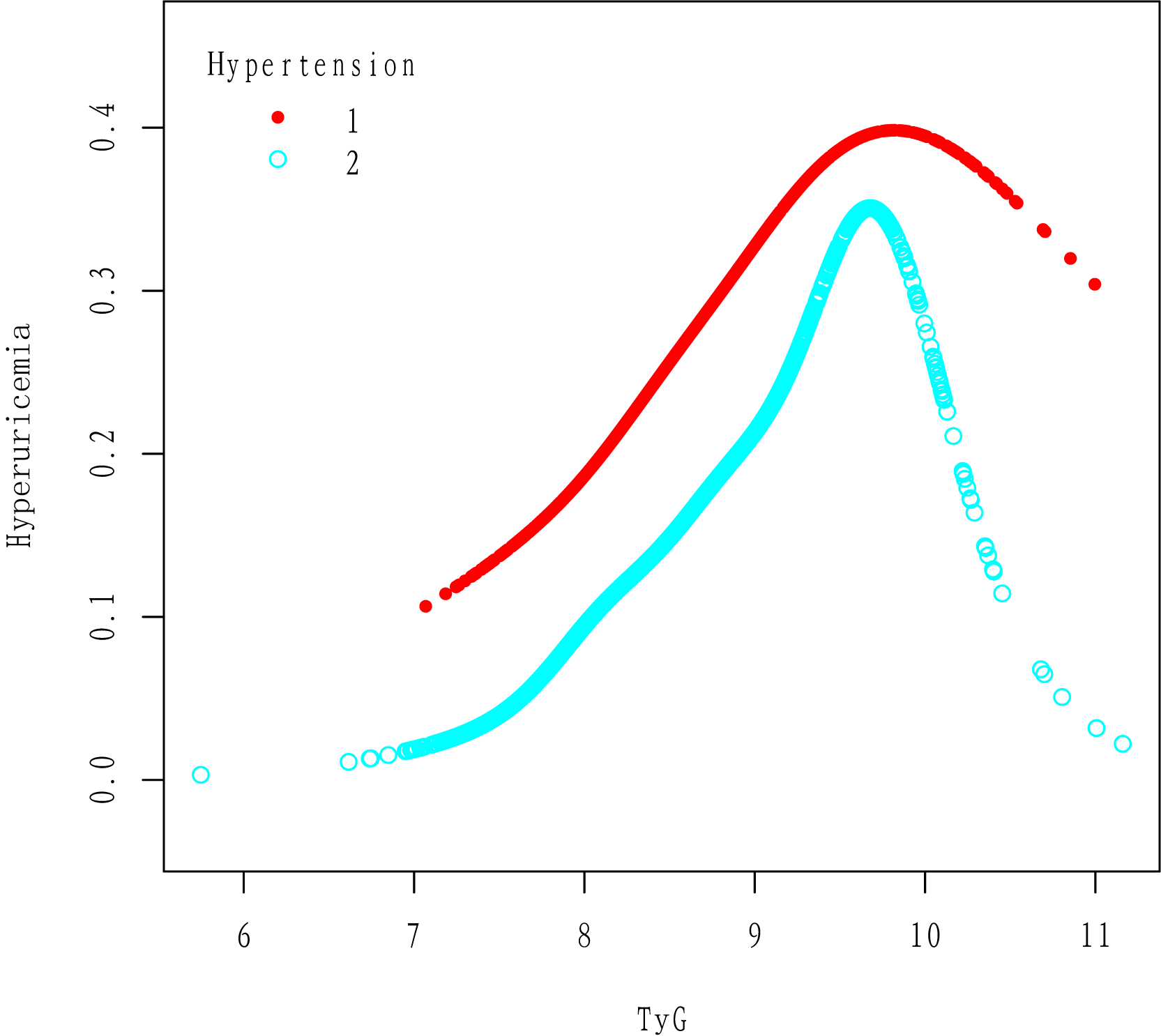

Supplement: Supplementary file 3 — Supplementary Material 3 [file 12944_2024_2146_MOESM3_ESM.png]

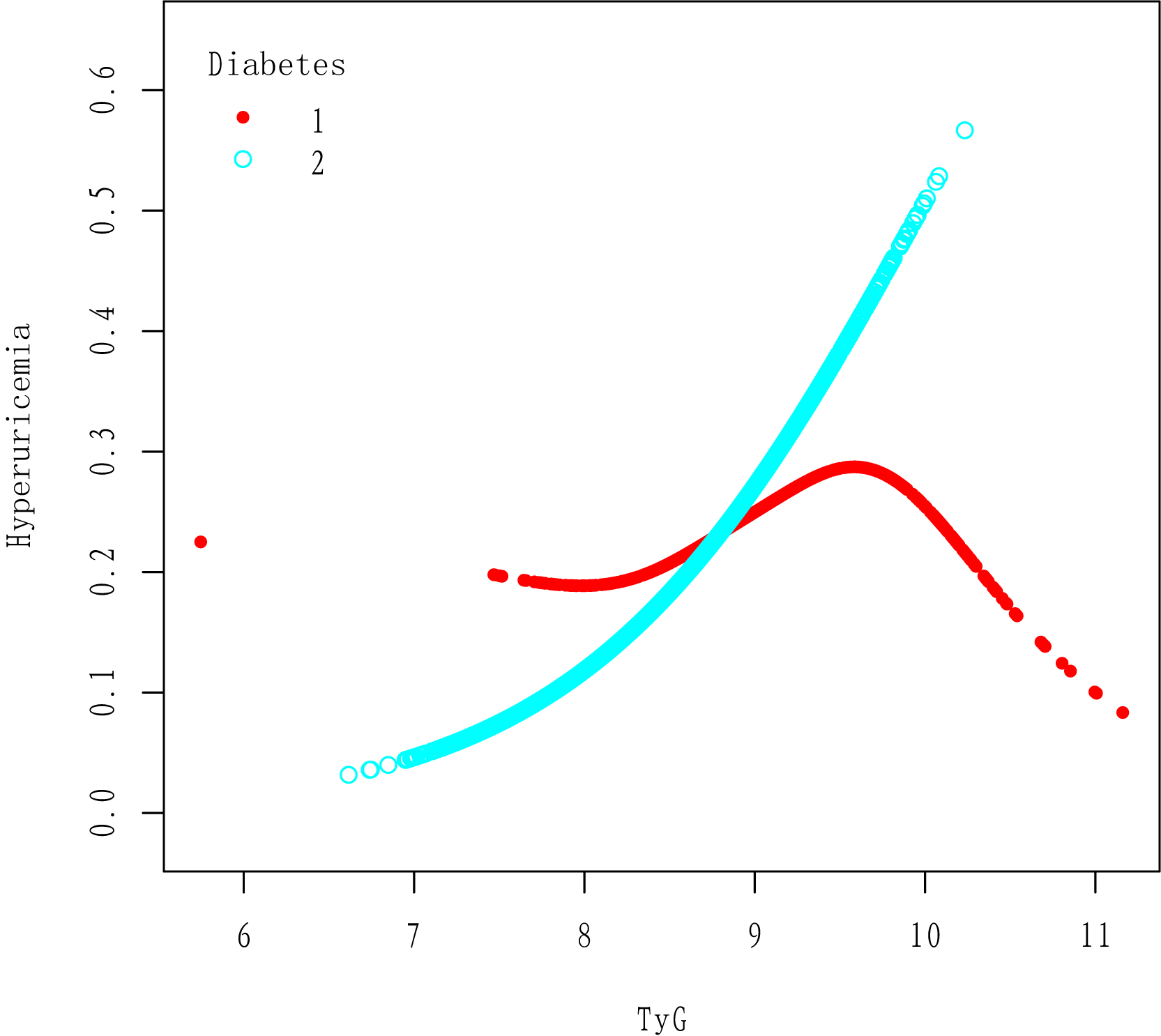

Supplement: Supplementary file 4 — Supplementary Material 4 [file 12944_2024_2146_MOESM4_ESM.png]
